# From H_2_O_2_ to OH: A First-Principles Investigation of the Heterogeneous Fenton-Like Reaction

**DOI:** 10.1021/acsomega.6c05647

**Published:** 2026-06-25

**Authors:** Basil Raju Karimadom, Dan Meyerstein, Amir Mizrahi, Haya Kornweitz

**Affiliations:** † Chemical Sciences Department and the Radical Reactions Research Center, 42732 Ariel University, P.O.B. 3 Ariel, Ariel 40700, Israel; ‡ Chemistry Department, Ben-Gurion University of the Negev, Beer-Sheva 84105, Israel; § 155609 Nuclear Research Centre Negev, Beer-Sheva 84190, Israel

## Abstract

Heterogeneous Fenton and Fenton-like reactions have superior advantages over homogeneous Fenton or Fenton-like reactions for various advanced oxidation processes (AOPs). An in-depth analysis of the heterogeneous Fenton and Fenton-like reactions is necessary to address the challenges facing their widespread application. First-principles DFT calculations are employed to study the heterogeneous Fenton-like reaction using the pristine Fe(110) surface in an aqueous medium. Spin-polarized DFT calculations are used in place of traditional DFT calculations to address realistic electronic properties. The adsorption energies of *OH• formed on the surface after spontaneous H_2_O_2_ dissociation are calculated at various surface coverages. The electronic structural calculations reveal that the difference in *OH adsorption at higher coverage arises due to the destabilization of Fe–O bonding. Partial oxidation of Fe surface layers is observed at higher *OH coverage due to the charge transfer from the surface Fe atoms to the oxygens. Even though the surface deprotonation of *OH is highly exergonic, at larger *OH concentrations, the surface contains both *OH and *O^–^ on the surface. These results reveal that the Fe atoms are not fully oxidized in heterogeneous Fenton-like reactions using ZVI, unlike in homogeneous Fenton reactions. The *OH formed in the heterogeneous Fenton-like reaction is less reactive than OH­(aq), its reduction potential is only 0.15 V vs SHE (at pH 14), and its ability to degrade organic pollutants is decreased. Further studies on surface corrosion mechanisms and reactions on oxide/hydroxide layers are necessary for analyzing heterogeneous Fenton and Fenton-like reactions.

## Graphical abstract

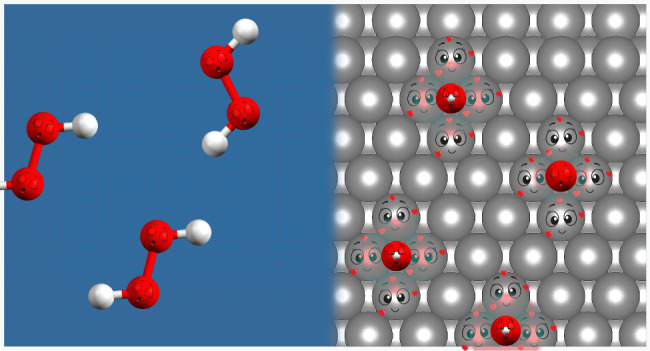

## 1 Introduction

Zero-valent iron (ZVI) is a strong reducing agent that is used for the effective removal of organic, inorganic, and radioactive pollutants.^1–8^ ZVI is widely used for the treatment of contaminated groundwater. The primary advantage of ZVI over other materials is its direct applicability to both soil and groundwater.^9^ In recent years, nanoscale ZVI (nZVI) has been replacing ZVI in the advanced oxidation processes (AOPs) due to its larger surface area and higher reactivity.^6,10^ Although many zerovalent state metals, such as Zn^0^, Sn^0^, and Al^0^,^11–13^ are used in the efficient treatment of contaminated groundwater, the economic advantage possessed by Fe^0^ has increased the research interest in it in recent decades.^6,12^ However, all these metals are covered with an oxide layer; therefore, it is required to study whether the zerovalent metal is involved in the heterogeneous Fenton reaction or the oxides that cover this metal. In this research, we focus on ZVI, and a subsequent study focusing on metallic oxides is planned.

The Fenton and Fenton-like reactions are widely studied AOPs used for wastewater treatment.^14–18^ The homogeneous Fenton reactions suffer from major disadvantages, such as the need for the removal of iron sludge, pH dependency, and the requirement for large concentrations of Fe^2+^.^12,19,20^ Therefore, the research has shifted toward developing heterogeneous catalysts for Fenton reactions. Clays, metal-exchanged zeolites, metal oxides, metal-exchange resins, etc., have been tested so far.^21,22^ The use of nZVI has gained more interest as it reduces the disadvantages associated with the homogeneous Fenton reactions, even though the reactions are slower.^12^

The reaction mechanisms of the heterogeneous Fenton and Fenton-like reactions with ZVI and nZVI are still not completely investigated. The most accepted oxidation mechanism involves two separate reaction channels: (a) from the iron ions released into the solution and (b) through the reaction between the solute and surface species.^6,10,12^

During adsorption, the p*K*_a_ of the adsorbates changes significantly from their homogeneous p*K*_a_ value.^23–25^ A recent study has shown that the surface p*K*_a_ (*p*K*_a_) of weak acids shifts significantly toward lower values upon adsorption on noble metal surfaces.^26^ The OH•, which is the major product of the Fenton reactions, has an aqueous p*K*_a_ of 11.8. A recent study has shown that the *p*K*_a_ of *OH on various noble metal surfaces drops significantly from the aqueous value, resulting in the deprotonation of *OH• into *O^–^ on the surfaces of Au^0^ and Pt^0^.^27^

In this study, we investigate the surface reaction channels of the heterogeneous Fenton-like reaction on the Fe(110) surface. The Fe(110) surface is chosen as various experimental and computational studies have reported the Fe(110) surface as the active surface for oxidation reactions.^28–30^ This study aims to understand the reaction mechanisms of the heterogeneous Fenton-like reaction and the factors affecting the surface reactions. The influencing factors, such as surface deprotonation and the effect of coadsorption, are being studied for the first time. A realistic knowledge of the heterogeneous Fenton and Fenton-like reaction mechanisms is necessary for the widespread application of the reaction and for the design of catalysts for other Fenton-like reactions.

## 2 Computational Details

The periodic DFT calculations were performed using the Vienna Ab-initio Simulation Package (VASP)^31^ with the GGA-PBE^32^ exchange-correlation functional. The PBE functional was chosen for this study because it has shown reliable results in experimentally validated studies of transition-metal surfaces for various reactions and has also been used for the Fe(110) surface.^33–36^

The ion core is modeled using the PAW^37^ pseudopotential, and the valence electrons are described using a plane-wave cutoff energy of 400 eV. The DFT-D3 dispersion correction with the BJ damping^38^ function is used to describe the long-range van der Waals interactions. The solvent effect is introduced using VASPsol,^39^ an implicit-electrolyte solvation model with the bulk dielectric constant value for water (78.4).

The Fe(110) surfaces are modeled with six layers of a metal slab, with each layer consisting of 8 Fe atoms, and a vacuum distance of 16 Å is kept between the slabs in the *z*-direction to avoid unwanted interactions. The (110) surface is considered the most common facet studied for the oxidation and oxygen adsorption reactions on Fe^0^.^29,40^ The Brillouin zone integrations are performed using the Monkhorst-Pack^41^ 8 × 8 × 1 k-point mesh. The spin-polarized calculations were performed with the initial on-site magnetic moment values of 2.1 μ_B_ (Fe) and 1.0 μ_B_ (O and H). The geometry optimizations are performed until the total energy difference between two successive iterations is below 1 × 10^–5^ eV. The SCF convergence is improved using the AMIX and BMIX parameters with values of 0.1 and 0.0001, respectively. The phonon calculations were performed to obtain the zero-point vibration energies (ZPVE) with a step width of 0.01 Å using the harmonic oscillator approximation. The transition state (TS) calculations are performed using the climbing Nudged Elastic Band (cl-NEB)^42^ method by considering five images between the initial and final states. The frequency analysis is performed on each initial and final state before NEB analysis to confirm that each geometry is a true ground state (no imaginary frequency). The Density of States (DOS) calculations were performed using tetrahedron smearing with the Blöchl corrections, and the results are postprocessed using VASPKIT.^43^ The Crystal Orbital Hamilton Populations (COHP) analysis was performed using the LOBSTER^44,45^ suite.

The counterion (CI) method^46^ is used for the calculations of charged systems, and the charge analysis is performed after each calculation using the Bader^47^ charge analysis program. The 3D visualization package VESTA^47^ is used to visualize the calculated structures and the charge density difference.

The adsorption energies (*E*_ads_) are calculated using Equation 1;

1 Eads=GX*−G*−GX(aq)

where X is the species adsorbed on the metal surface (*), G* is the free energy of the Fe(110) surface, $G_{X_{\left(\mathrm{aq}\right)}}$ is the free energy of isolated molecule X and GX* is the free energy of the adsorbed system of X on the Fe(110) surface. The free energy value *G* is defined as

2 $$G=E_{\mathrm{DFT}}+\mathrm{ZPVE}-\left(T\times S\right)$$

where *S* is the entropy, and *T* is the temperature (298.15 K). The entropy value is calculated using the VASPKIT interface.^43^ The negative (positive) *E*_ads_ values signify strong (weak) adsorption. The * symbol is used throughout this manuscript to mention that the species is adsorbed on the surface, and the solution-phase species are duly mentioned with “aq” subscript.

## 3 Results and Discussion

The adsorption of H_2_O_2_ on the Fe(110) surface is the primary interest of studying heterogeneous Fenton-like reactions. Our previous study on noble-metal surfaces showed that the H_2_O_2_ molecules are weakly physisorbed on the surface with positive adsorption energy values.^27^ However, the SCF simulations for the adsorbed structure of H_2_O_2_ resulted in the dissociation of the H_2_O_2_ into ∗OH species on the surface. The structural dissociation is also observed at higher H_2_O_2_ coverages

3 H2O2aq+*→2OH*

On the Fe(110) surface, the adsorbed geometry of H_2_O_2_ is not observed, and it undergoes spontaneous homolytic O–O cleavage, resulting in ^∗^OH on the surface via reaction 3. The reaction is highly exergonic, with a Δ*G* ^0^ value of −4.38 eV, and the cl-NEB analysis indicates that the reaction proceeds without any activation energy barrier.

The *OH formed on the surface adsorbs at the long bridge site of the Fe (110) surface (Figure 1) through the oxygen atoms, and the hydrogen atom is aligned perpendicular to the surface. The adsorbed geometry and the adsorption site are in line with previously reported studies.^28,30^ The *OH adsorption is highly exergonic (*E*_ads_ = −3.38 eV) with a charge transfer (C.T.) of 0.63e̅ from the Fe surface to the *OH. The diffusion energies for the *OH on the surface are calculated using the cl-NEB method. The *OH has a small diffusion energy of 0.24 eV, which enables *OH to diffuse on the Fe surface.

**Figure 1:**
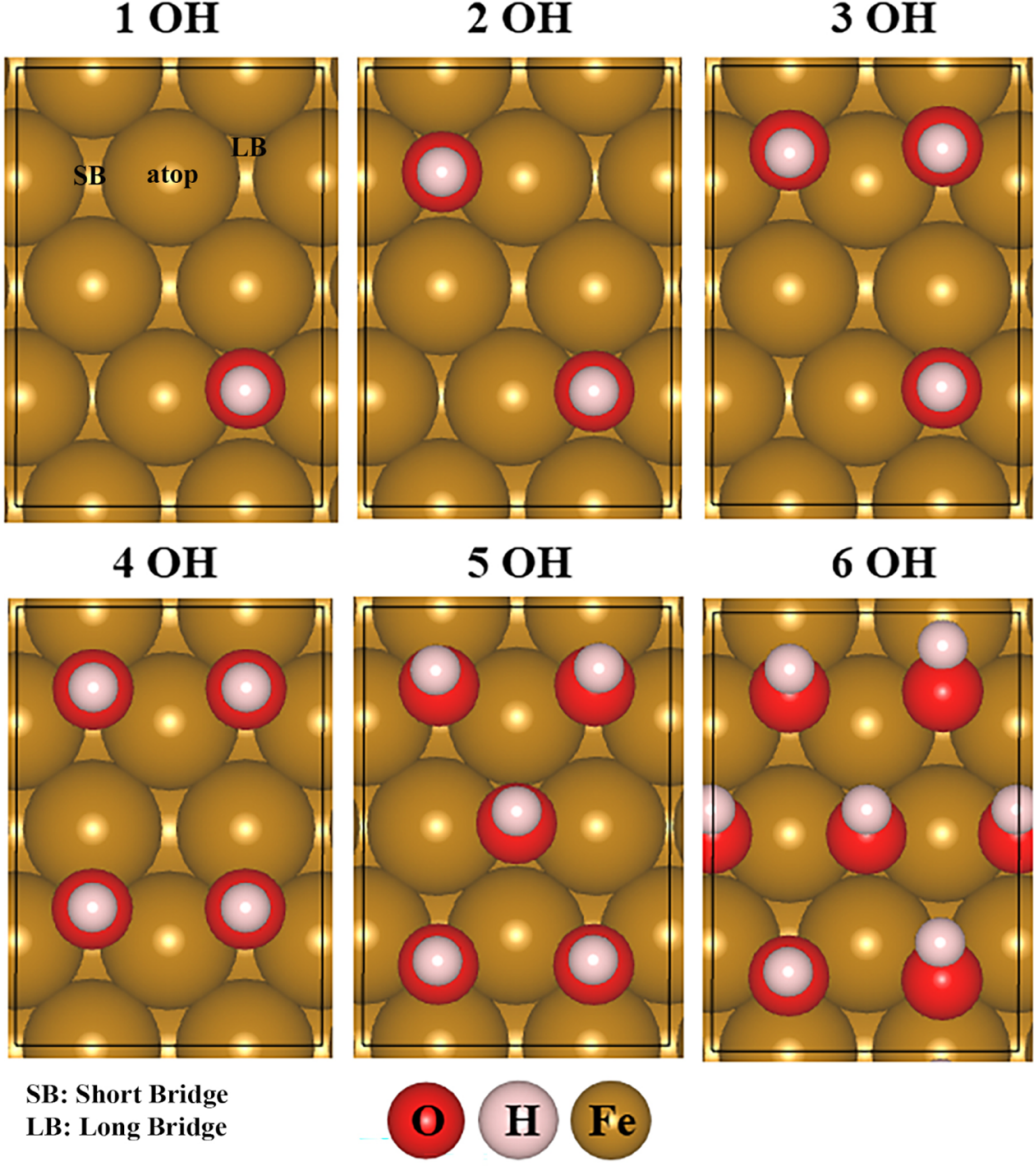
Optimized geometries of *OH adsorption at different surface coverages on the Fe(110) surface.

### 3.1 Surface Coverage of *OH

According to reaction 3, *OH is formed on the surface, and at higher concentrations of H_2_O_2_, the amount of OH formed also increases linearly. The effect of increased surface coverage of *OH on the Fe surface is studied in this section.

The equation for the average adsorption energy (*E*_ads_) per one hydroxyl is modified for the *OH at different coverage as

4 $$E_{\mathrm{ads}}=\frac{1}{n}\left(E_{*n{\mathrm{OH}}^{•}}-E_{*}-nE_{OH_{\mathrm{aq}}}\right)$$

where *n* is the number of *OH adsorbed on the surface. Along with *E*_ads_, Δ*G*_ads_ is also considered for the addition of each new *OH• to the surface. The Δ*G*_ads_ is defined as follows in Equation 5

5 $$\Delta G_{\mathrm{ads}}=G_{*nOH^{•}}-G_{OH_{\mathrm{aq}}^{•}}-G_{*\left(n-1\right)OH^{•}}$$

The free energy, *G*, of each structure is obtained using Equation 2. Each new *OH is added to the long-bridge site until further addition results in the distortion of the structure or the formation of new species during the SCF cycle. The obtained *E*_ads_ and Δ*G*_ads_ values are tabulated in Table 1. The optimized geometries are given in Figure 1, and the structural parameters are provided in the SI. The surface of Fe(110) is rectangular, with a surface area of 43.81 Å^2^, and from herein, *x*/8 ML represents the coverage of *x* number of OH per 43.81 Å^2^ surface area of Fe.

**Table 1:** Adsorption Energy (*E*_ads_), Free Energy of Adsorption (Δ*G*_ads_), Total Charge Transfer (C.T.), and Average Charge Transfer on Each OH from the Surface at Different Surface Coverages of OH• on the Fe(110) Surface

No. of *OH	*E*_ads_ (eV)	Δ*G*_ads_ (eV)	Total C.T. (e̅)^a^	C.T./OH (e̅)
1	–3.38	–3.38	–0.63	–0.63
2	–3.49	–3.59	–1.29	–0.64
3	–3.33	–3.01	–1.96	–0.65
4	–3.23	–2.92	–2.64	–0.66
5	–3.15	–2.86	–3.27	–0.65
6	–3.06	–2.60	–3.92	–0.65

a A negative C.T. represents charge transfer to the adsorbate from the surface and vice versa.

The maximum number of *OH molecules that can be adsorbed on the surface is found to be 6. When the seventh OH^•^ is added to the surface, the structural optimization leads to the destruction of the adsorbates on the surface. Such optimization results in the formation of species such as H_2aq_, *O, and H_2_O_aq_ in the system with different SCF attempts (Figure S1). Therefore, the maximum coverage of *OH on the Fe(110) surface is 6/8 ML.

The *E*_ads_ and Δ*G*_ads_ of *OH are reduced with the increase in coverage on the Fe(110) surface. Even at the highest coverage, the adsorption energies are large, which signifies the strong adsorption of *OH on the Fe surface. The strong adsorption of *OH is accompanied by a large C.T. from the Fe surface to *OH. Unlike in the homogeneous medium or single-atom reaction,^16,18^ the electron transfer to the oxygen atom of *OH is not observed from a single Fe atom; instead, the C.T. is observed from four of the neighboring Fe atoms on the long-bridge site. The overall C.T. between *OH and the Fe surface is given in Table 1, and the charge transfer between the Fe surface atoms and *OH is analyzed using a charge-density difference plot. The charge-density difference (CDD) plot for the selected coverage of *OH is given in Figure 2, and complete files are provided in the SI.

**Figure 2:**
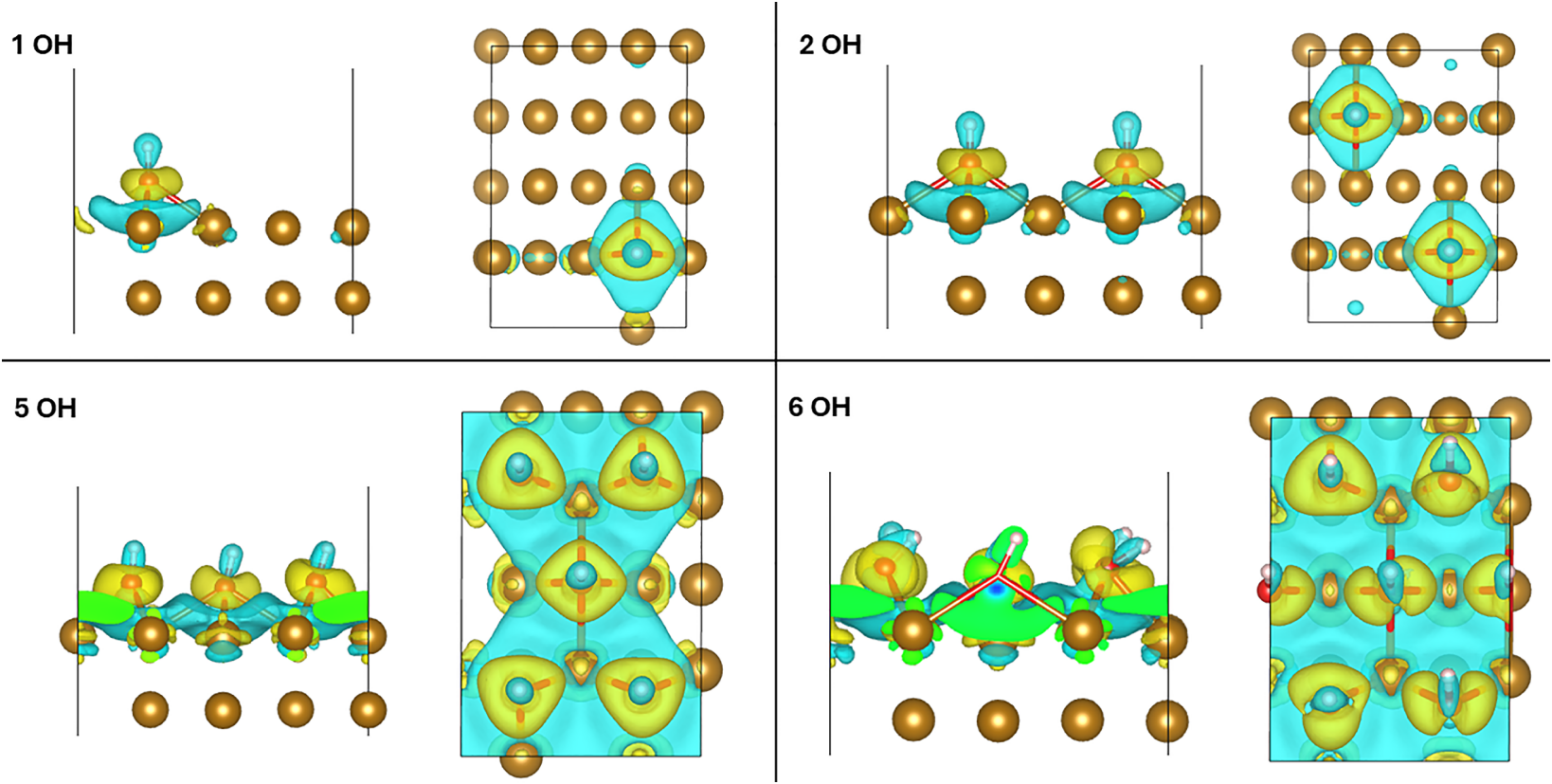
Side and top views of the charge-density difference (CDD) plot of *OH• adsorption at different coverages on the Fe(110) surface.

In the CDD plot, the electron-accepted region is shaded in yellow, and the electron-donated region is shaded in cyan. The oxygen atom accepts electron density from both the H atoms and the surface. As the surface coverage of *OH increases, the total charge transfer from the Fe surface increases linearly (Figure S2). The CDD plot implies that at different coverages, only the surface Fe atoms undergo oxidation, and the Fe atoms in the inner layers are not affected by the adsorption. At the highest coverage (6/8 ML), the highest coordination number of Fe is three *OH. At the highest coverage, the total charge transferred from the surface is −3.92e̅. So, unlike in a homogeneous Fenton reaction, where Fe^2+^ is oxidized to Fe^3+^,^18^ on a heterogeneous Fe surface, the Fe atoms are less oxidized during the reaction of H_2_O_2_. In the homogeneous reaction, each Fe atom loses one electron, while in the heterogeneous process, eight Fe atoms transfer 4­(e̅), half an electron on average per Fe atom. This result is in line with the reduction potentials of Fe^2+^ and Fe, *E* ^0^(Fe^3+^/Fe^2+^) = −0.77 V, and *E* ^0^(Fe^2+^/Fe^0^) = −0.44 V. It cannot be ruled out that at a large coverage of Fe^2+^ is released over time and/or that an oxide/hydroxide layer is formed.

#### 3.1.1 Effect of Spin-Polarized Calculations

Fe has unpaired 3d electrons and gives a net magnetic moment to the material. So, the exclusion of spin polarization will result in unphysical nonmagnetic states for Fe, and it will result in incorrect total energies and other properties that are studied. Therefore, unlike our previous studies of H_2_O_2_ surface reactions, spin-polarized calculations were performed in the current work. However, the nonspin-polarized calculations were also performed on the same systems to understand which physical quantities are affected by the spin-polarized calculations. In Figure S3, the DFT energy (*E*_DFT_), zero-point vibration energy (ZPVE), and entropy contribution (*T* × *S*) are plotted for spin- and nonspin-polarized calculations. While the ZPVE and *T* × *S* values are less influenced by spin polarization, the *E*_DFT_ values are affected significantly (>5 eV). The differences in the DFT electronic energy are larger for the pristine Fe surface than for the adsorbed surfaces; therefore, the adsorption energies for the spin-polarized calculations are smaller. The spin-polarized calculation also affects the geometry of the surface, as the nonspin-polarized calculations converge to a different arrangement for the Fe(110) surface (Figure S3). The hollow sites in the nonspin-polarized structure become long-bridge sites in the spin-polarized calculations. The magnetization is artificially removed for Fe in the nonspin-polarized calculations, causing the d-states of Fe to cross the Fermi level into the antibonding level (Figure S8). With spin polarization, the d-states are occupied below the Fermi level, and the splitting of the d-band is considered. The splitting of the d-states affects calculations of properties such as adsorption energy, bond strength, etc. In Table S2, the *E*_ads_ of *OH at different coverages is tabulated for spin polarized and nonspin-polarized calculations. The nonspin-polarized calculations overestimate the *E*_ads_, even though the trend is similar to that of the spin-polarized calculations. Additionally, the surface distortion of *OH is observed only at 8/8 ML coverage in the nonspin-polarized calculation. Therefore, the exclusion of spin polarization in calculations involving magnetic materials, such as Fe, or for radicals or any other open-shell species, will yield qualitatively incorrect results.

#### 3.1.2 Electronic Structure Calculations

The in-depth bonding analysis of *OH on the Fe surface is further studied by using various electronic structural calculations.

##### 3.1.2.1 Density of States (DOS) Calculation

The DOS calculations are the most common analysis tool used for understanding the electronic distribution in materials. The spin-polarized calculation provides the DOS data for both spin orientations of the Fe and the *OH. The p-DOS of Fe up-spin d-states with O_p_ is given in Figure 3, and the down-spin data is plotted in Figure S4. As Fe is a ferromagnetic material, the up-spin d-states of Fe are located below the Fermi level, and the down-spin states are occupied mainly above the Fermi level. The band center (ε) energy difference has similar values for down and up of O_p_ with Fe_d_ (Table S1). The extent of overlap between down-spin O_p_ states and up-spin Fe_d_ states is larger than that between up-spin O_p_ states and Fe_d_ states. At higher coverage, the O_p_ states spread more over the Fe_d_ states, but simultaneously, the O_p_ states also move away from the Fermi level. So, the overall effect will weaken the Fe–O binding at higher coverage.

**Figure 3:**
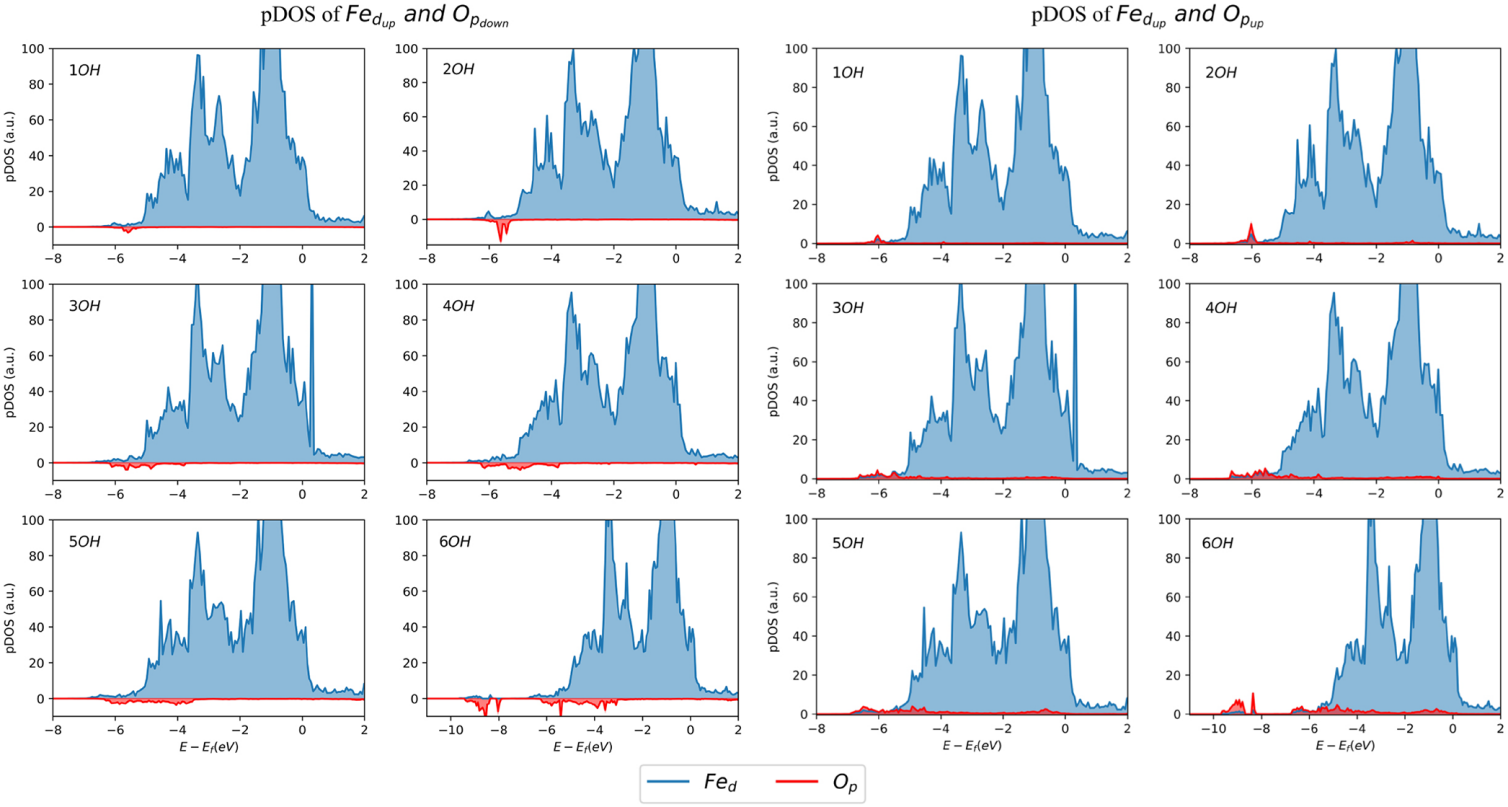
Normalized partial density of states (pDOS) of *OH adsorption at different coverages on the Fe(110) surface.

The metal d-band center (ε_d_) model is also used as a reliable indicator for analyzing the metal–adsorbate binding.^48^ The ε_d_ of Fe at different coverages of *OH, and for the plain surface, is plotted in Figure S5. As the number of *OH on the surface increases, the ε_d_ shifts away from the Fermi level. Although the changes are small (<0.2 eV), the ε_d_ values provide insight into the weakening of Fe–O binding at high coverage.

##### 3.1.2.2 Crystal Orbital Hamilton Population (COHP) Analysis

COHP analysis is an effective tool for analyzing the bonding between two atoms.^44,49^ Since the OH• is adsorbed on the Fe surface through the oxygen atom, the COHP is plotted for the Fe–O bond for the *OH• adsorption at different surface coverages (Figure 4). Unlike DOS, the COHP analysis clearly distinguishes the bonding and antibonding states. In the COHP graph (Figure 4), the positive COHP region represents bonding states and the negative region represents the antibonding states. At higher coverage, the destabilization of Fe–O binding occurs due to the increased occupancy of antibonding states. As a combined effect of surface crowding, more electrons are pushed toward the antibonding state, resulting in the weakening of the Fe–O binding at higher coverage.

**Figure 4:**
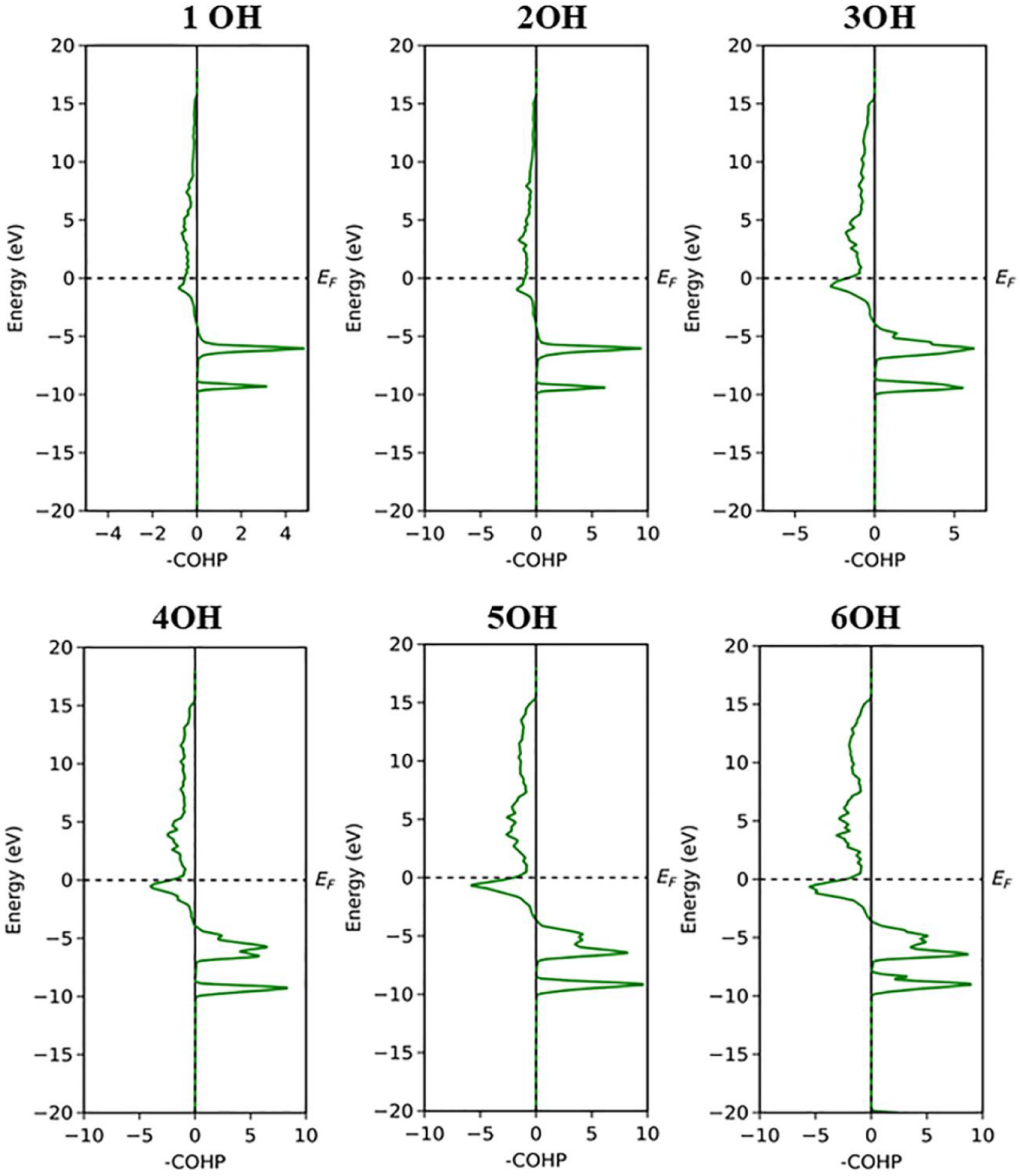
COHP plot of the Fe–O bonding pair at different surface coverages of *OH• on the Fe(110) surface.

To further understand the bonding, we consider integrated COHP (ICOHP) analysis. The ICOHP analysis is performed to study one of the*OH• adsorbed on the surface. The Fe–O ICOHP values are plotted for the same Fe–O bonds at different coverages to map how the bond strength varies with coverage (analyzed Fe–O bonds are labeled in Figure S6). The ICOHP values are plotted in Figure 5(a). The more negative ICOHP values signify strong binding, and the increase in values (less negative) signifies weakening of the bonding. The *OH is initially adsorbed on the long bridge site (Figure 1) on the Fe (110) surface. Due to this, the strongest interaction of oxygen is between Fe2 and Fe1. This bond strength weakens monotonically with the surface coverage. The bond distance (Figure 5(b)) is plotted alongside the ICOHP, and both graphs exhibit similar trends. The bond between Fe6 and Fe8 with O does not follow a trend as the OH moves from one long bridge site to the other at high surface coverage. At the highest coverage (6/8 ML), the *OH• moves further away from Fe8, and it binds to the surface only through three Fe atoms instead of four at the lowest coverage. So, at the highest coverage, each Fe atom on the surface is bound to three O atoms, and each O is also bound to three Fe atoms.

**Figure 5:**
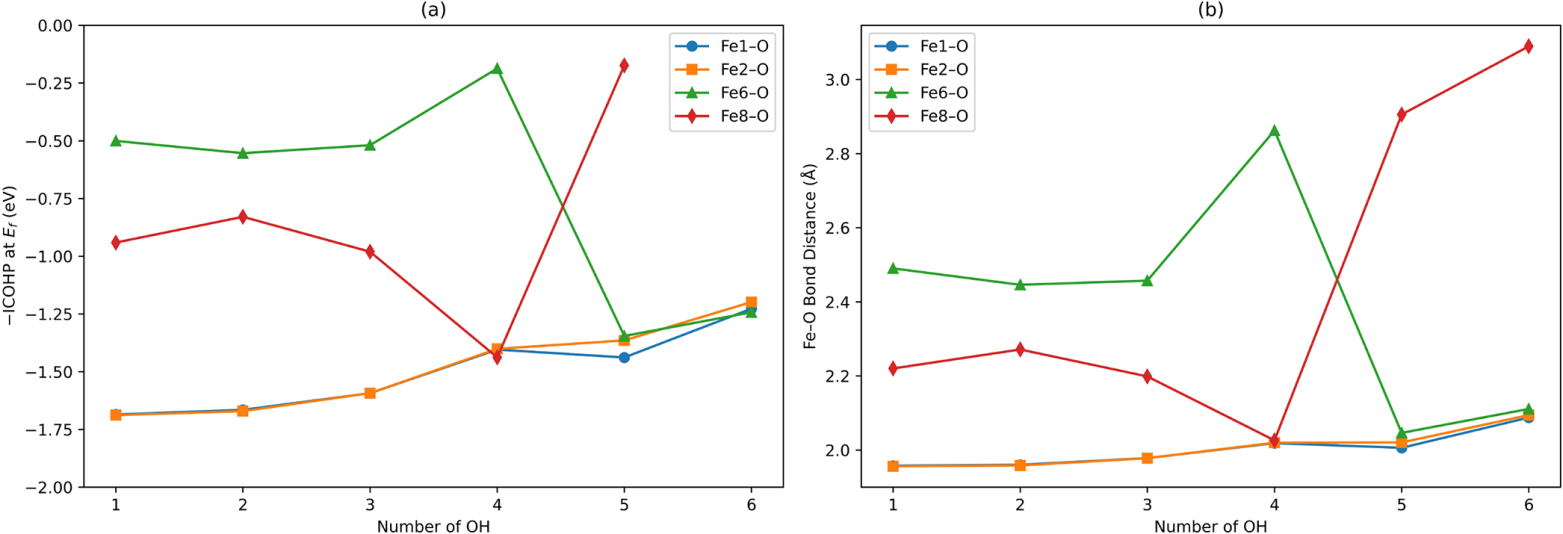
ICOHP values of various Fe–O bonds (a) and bond distances of various Fe–O bonds (b) at different surface coverages of *OH• on the Fe(110) surface.

### 3.2 Surface Reactions of *OH

#### 3.2.1 Adsorption Energies of Other Species

Before studying various surface reactions of hydroxyl on the surface, the adsorption of various species relevant to those reactions is considered. The properties, such as the adsorption site, adsorption energy, and charge transfer of species involved in the reactions, are provided in Table 2. The optimized geometries of these species are given in Figure S7.

**Table 2:** Adsorption Energy (*E*_ads_), Charge Transfer (C.T.), and Distance between the Surface and the Adsorbate for Various Adsorates on the Fe(110) Surface

Adsorbate	Adsorption Site	*E*_ads_ (eV)	C.T. (e̅)^a^	Distance from Fe (Å)
OH^–^	Long bridge	–1.63	0.30	1.99
H_2_O	Atop	0.26	0.08	2.09
O·	Long bridge	–6.59	–1.14	1.86
O^–^	Long bridge	–5.55	–0.18	1.87
H·	Long bridge	–2.71	–0.45	1.77
H^+^ ^b^	Long bridge	13.75	–1.42	1.77

a A negative C.T. represents charge transfer to the adsorbate from the surface and vice versa.

b Cl^–^ counterion is used.

The species are adsorbed mainly through the long-bridged site on the Fe(110) surface. The *OH^–^ has lower adsorption energy than the *OH•, and the charge transfer is observed from the adsorbate to the surface. Only H_2_O is adsorbed on the atop site; it adsorbs through the oxygen atom, with the two hydrogens oriented parallel to the surface. These adsorption geometries are similar to those reported in previous studies.^50,51^ The adsorption energy of H_2_O is positive, with almost no C.T., indicating that the adsorption of water is physisorption and not chemisorption. Due to the large solvation energy of H^+^, the adsorption of H^+^ is highly endergonic; meanwhile, the adsorption of H• is highly exergonic on the surface.

The adsorption of *O• and*O^–^ is stronger than that of the other species. The strong adsorption energy of *O• is mainly due to the large extent of charge transfer from the surface. The adsorbed *O• becomes *O^–^ at the surface due to the charge transfer. Tan et al. have previously reported the C.T. from the Fe to the O atom during adsorption.^29^

#### 3.2.2 Surface Reactions

The initial adsorption of H_2_O_2_ will result in the formation of *OH on the Fe(110) surface, and thus, this section discusses the further reactions of the *OH

6 2OH∗→O∗+H2O∗ΔG0=0.41eV

The reaction between two *OH• is an endergonic process on the surface due to the strong *E*_ads_ of *OH•·on the surface. The reaction of *OH•· with a preadsorbed *H is also an endothermic process due to the strong adsorption of the radicals

7 OH∗+H∗→H2O∗ΔG0=0.93eV

The *OH• can also undergo a homolytic cleavage of the O–H bond on the surface as

8 OH∗→H∗+O∗ΔG0=−0.52eV

The reaction is highly exergonic on the surface, as the two formed radicals are strongly adsorbed on the surface. The cl-NEB analysis was performed to calculate the activation energy (*E*_a_) barrier for the reaction, and the result obtained was 0.80 eV. The large *E*_a_ for the *O–H homolytic cleavage implies the strength of the O–H bond. *E*_a_ was also recalculated at different surface coverages of *OH, from 1/8 ML to 3/8 ML, to account for the dependency of the surface coverage on the *O–H bond cleavage. *E*_a_ only showed a small shift, from 0.80 to 0.73 eV at 3/8 ML coverage. Thus, the *O–H homolytic cleavage is not preferred on the Fe(110) surface, even at high coverage of *OH.

At high concentrations of H_2_O_2_, aqueous H_2_O_2_ is present in the system. The aqueous H_2_O_2_ can react with the adsorbed *OH on the surface as follows:

9 H2O2(aq)+OH∗→H2O∗+O∗+OH∗ΔG0=−3.97eV

The reaction is highly exergonic, but the cl-NEB analysis reveals that it is a multistep process, initiated by the surface dissociation of H_2_O_2_ when it approaches the Fe surface (Reaction 3). The formation of *H and *OH on the surface is through the reaction with two *OH, and it has been shown that the reaction is endergonic on the surface (Reaction 6). So even though overall Reaction 8 is exergonic, it will not proceed due to the reaction kinetics.

We have recently shown that the surface deprotonation of *OH• is highly exergonic on the surfaces of Pt and Au. The surface p*K*_a_ (*p*K*_a_) of *OH• shifted toward lower values from the aqueous p*K*_a_ values (aq. p*K*_a_ = 11.8). The *p*K*_a_ of *OH• on the Fe(110) surface is calculated using the reported equation.^26^ The calculated *p*K*_a_ for *OH/*O•^–^ on the Fe surface is −24.70 (Δ*G* ^0^ = −1.46 eV). Similar to previous studies, the *OH deprotonation is highly exergonic on the surface. The heterogeneous *O–H cleavage is a barrierless reaction due to the large solvation energy of H^+^, and H^+^ is released into the aqueous medium. The reaction is as follows:

10 OH∗→O−∗+H(aq)+

As the adsorption energies are influenced by the surface coverage of adsorbents, and the *p*K*_a_ calculations are functions of adsorption energies, the effect of surface coverage is studied for Reaction 10. At the 2/8 ML coverage, the *p*K*_a_ of *OH• is increased to −2.40. The shift in *p*K*_a_ arises due to the change in *G*_ads_ of O^–^ at 2/8 ML coverage. While adsorbing alone, *O^–^ has an adsorption energy of −5.55 eV, whereas at a 2/8 ML coverage, the average adsorption energy reduces to −4.33 eV. So, due to these changes, the *p*K*_a_ shifts to higher values at 2/8 ML coverage. Due to computational limitations in calculating highly charged species, we cannot consider the complete deprotonation of *OH• at higher coverage than 2/8 ML.

In order to compute the effect of surface coverage, the deprotonation of *OH• is calculated by considering the excess *OH• on the surface as a coadsorbate. Instead of complete deprotonation, only one of the *OH• molecules is considered to undergo deprotonation. The new *p*K*_a_ values are tabulated in Table 3. This also shows the increase of *p*K*_a_ at higher surface coverage. But compared to the previous calculation, the change is lower. Even though the calculations are performed with the implicit solvation model, one H_2_O is explicitly added to the surface to calculate the effect of coadsorbed H_2_O on the *p*K*_a_ of *OH. Unlike *OH coadsorption, H_2_O coadsorption does not make significant changes to the *p*K*_a_ and for the deprotonation free energy. This result is expected, as there is almost no C.T. during the adsorption of H_2_O; thus, its adsorption does not affect the surface. So on the surface, both *OH and *O^–^ are obtained after the reactions. As the charge transfer to *O^–^ from the Fe is lower than to *OH, the Fe surface is less oxidized in the presence of *O^–^.

**Table 3:** Free Energies of Adsorption (*G*_ads_), *p*K*_a_, and Δ*G* ^0^ of Deprotonation of *OH•, with Different Coadsorbates

	*G*_ads_ (eV)			
Coadsorbate	OH•	O•^–^	*p*K*_a_ ^a^	Δ*G* ^0^ (eV)
None	–3.38	–5.55	–24.70	–1.46
1 OH	–3.59	–5.28	–16.76	–0.99
2 OH	–3.01	–4.72	–17.10	–1.01
H_2_O	–3.43	–5.52	–23.57	–1.39

a Homogeneous aqueous p*K*_a_ of OH is 11.8.

#### 3.2.3 Surface Methanol Oxidation

As the major application of Fenton reactions is for the degradation of organic pollutants, an example of surface oxidation on the Fe surface is considered. Methanol (CH_3_OH) oxidation generally proceeds with the hydrogen abstraction reaction to form the hydroxymethyl radical (CH_2_OH•) by the OH• radical. This reaction is energetically favored and fast in an aqueous medium.^52^ On the Fe(110) surface, CH_3_OH adsorbs through the oxygen atom at the atop site with an *E*_ads_ of 0.03 eV (Figure S9), while CH_2_OH adsorbs through both carbon and oxygen atoms with an *E*_ads_ of −1.34 eV (Figure S9). On the surface, the reaction is as follows:

11 CH3OH∗+OH∗→CH2OH∗+H2O(aq)ΔG0=0.29eV

Reaction 11 is endergonic on the surface, and reaction with aqueous CH_3_OH is more endergonic on the surface (Δ*G* ^0^ = 0.45 eV). So, contrary to the reaction in aqueous media, the degradation of methanol on the Fe surface is not plausible. This implies that although the heterogeneous Fenton-like reaction has advantages over the homogeneous Fenton reaction, it also has limitations for certain applications. Unlike aqueous media, on the plain surface, the radicals are stabilized by C.T. from the surface during the adsorption, and thus their reactivity is reduced. The reduced reactivity of *OH is demonstrated by its reduced reduction potential, 0.15 V vs SHE (the details of these calculations are given in SI Section 3), in comparison to 1.90 V^53^ in aqueous solutions at pH 14.0. Under experimental conditions, the oxide/hydroxide layer is formed on the surface, and the reactions on such oxide/hydroxide layers have to be studied further to elucidate the overall reaction mechanism of heterogeneous Fenton and Fenton-like reactions.

## 4 Concluding Remarks

The current study reports the surface reactions and properties of the heterogeneous Fenton-like reaction on the plain Fe(110) surface by using first-principles calculations. Unlike the noble metal surfaces, H_2_O_2_ adsorbs dissociatively as *OH at the long-bridge site on Fe. The dissociation is highly exergonic due to the strong adsorption energies of *OH, and charge transfer from the binding Fe atoms to adsorbed O follows the adsorption. The adsorption energy reduces with the increase in the surface coverage of *OH, and the maximum surface coverage of *OH on the Fe surface is 6/8 ML. The C.T. increases linearly with the surface coverage, and at maximum coverage, the Fe surface atoms undergo partial oxidation.

The weakening of Fe–O bonding at higher coverage is analyzed using electronic structural calculations. The pDOS and COHP analysis confirmed the bond weakening at higher coverage. At higher coverage, due to surface crowding, more electrons are pushed toward the antibonding states, which weakens the metal–oxygen bond. The *OH undergoes faster surface deprotonation at lower coverage over the O–H homolytic bond cleavage or other parallel surface reactions. The surface deprotonation reduces at higher surface coverage due to the presence of *O^–^ formed on the surface. Thus, the Fe surface will be covered with both *OH and *O^–^ after treatment with H_2_O_2_. Unlike the aqueous reactions, the degradation of methanol on the Fe^0^ surface is not initiated due to the strong adsorption of *OH. The reduction potential of *OH is only 0.15 V vs SHE; therefore, although *OH is formed in the heterogeneous Fenton-like reaction, *OH is less reactive than OH_(aq)_, and its ability to degrade organic pollutants is decreased. It seems that the Fenton reactions proceed mainly on the oxide layer and not on the plain surface. The importance of spin-polarized DFT calculations for magnetic materials like Fe is reported in this study. The exclusion of spin polarization results in quantitative errors in calculations of DFT energies and geometries of surfaces. The electronic calculations also validated the need for spin polarization, as its exclusion results in nonrealistic electronic density of states.

Although the current study elucidates the surface properties of heterogeneous Fenton-like reactions in aqueous media, the effect of explicit water molecules is not thoroughly considered. The effect of water molecules on the surface and the series of corrosion reactions observed on the Fe surfaces have to be studied. The current study is limited to Fe^0^, and these reactions should be further studied on nonzero-metal oxide/hydroxide surfaces. Further exploration of these is relevant for addressing the challenges associated with the applications of heterogeneous Fenton- and Fenton-like reactions in various AOPs.

## Supplementary Material



## Notes

The binding energies, redox potential, electronic states, and structural information of species on the Fe surface (PDF)

## ∥

CEITEC – Central European Institute of Technology, Masaryk University, 62500 Brno, Czechia
