# Supplementary material for: From H2O2 to OH: A First-Principles Investigation of the Heterogeneous Fenton-Like Reaction
Source: ACS Omega. 2026 Jun 25;11(26):39456–65. doi: 10.1021/acsomega.6c05647 (PMC13347345; doi:10.1021/acsomega.6c05647)
Supplement: Supplementary file 1 [file ao6c05647_si_001.pdf]

From H_2O_2 to OH: A First-principles Investigation of the Heterogeneous Fenton-Like Reaction

Basil Raju Karimadom,[1,2] Dan Meyerstein,[1,2] Amir Mizrahi[3] and Haya Kornweitz[1*]*

[1] Chemical Sciences Department and The Radical Reactions Research Center, Ariel University, P.O.B. 3 Ariel, 40700, Ariel, Israel.

[2] Chemistry Department, Ben-Gurion University of the Negev, Beer-Sheva 84105, Beer-Sheva, Israel

[3] Nuclear Research Centre Negev, Beer-Sheva 84190, Beer-Sheva, Israel

Email: [*] basilkarimadom@gmail.com, danm@ariel.ac.il, amirmiz@post.bgu.ac.il, hayak@ariel.ac.il

Supporting Information

1. Tables

Table S1: The band center (ε) difference (eV) between Fe_d and O_p with different spin orientation at different surface coverage of $OH^{\cdot}$.

No. of $OH^{\cdot}$	$\varepsilon_{d(up\ spin)} - \varepsilon_{p(down\ spin)}$	$\varepsilon_{d(up\ spin)} - \varepsilon_{p(up\ spin)}$	$\varepsilon_{d(down\ spin)} - \varepsilon_{p(down\ spin)}$	$\varepsilon_{d(down\ spin)} - \varepsilon_{p(up\ spin)}$
**1**	-3.68	-3.621	-5.908	-5.849
**2**	-3.815	-3.747	-6.067	-5.999
**3**	-3.331	-3.526	-5.511	-5.706
**4**	-3.309	-3.309	-5.519	-5.519
**5**	-4.284	-3.423	-6.545	-5.684
**6**	-3.077	-3.27	-5.368	-5.561

Table S2: The adsorption energy (E_{ads}) (eV) of $OH^{\cdot}$ at different coverage on the Fe(110) surface with and without spin-polarisation.

Adsorbate	w/o Spin	With Spin
1 OH	-4.28	-3.38
2 OH	-4.19	-3.49
3 OH	-4.15	-3.33
4 OH	-4.08	-3.22
5 OH	-3.75	-3.15
6 OH	-3.63	-3.06
7 OH	-3.34	-

2. Figures

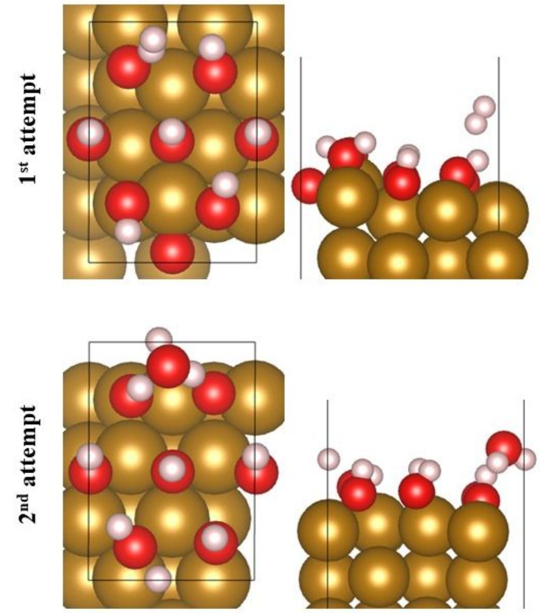

Figure S1: The structures after optimisation of 7 OH· on the Fe(110) surface.

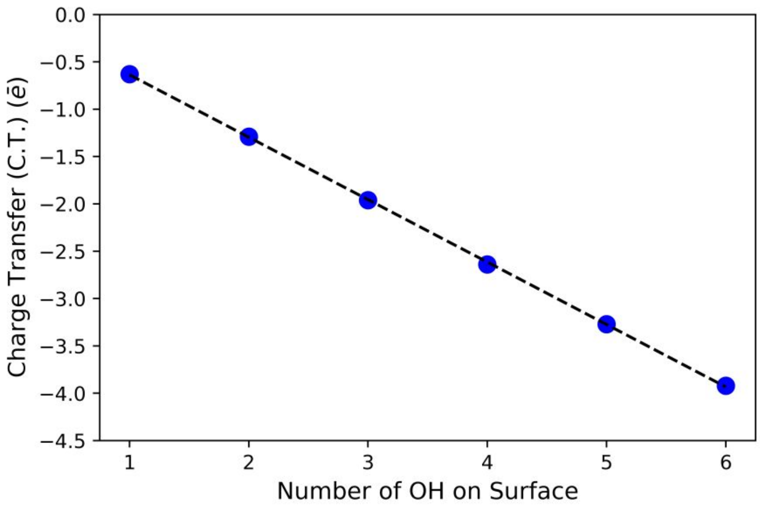

Figure S2: Plot of total charge transfer from the Fe(110) surface to *OH· at different coverage.

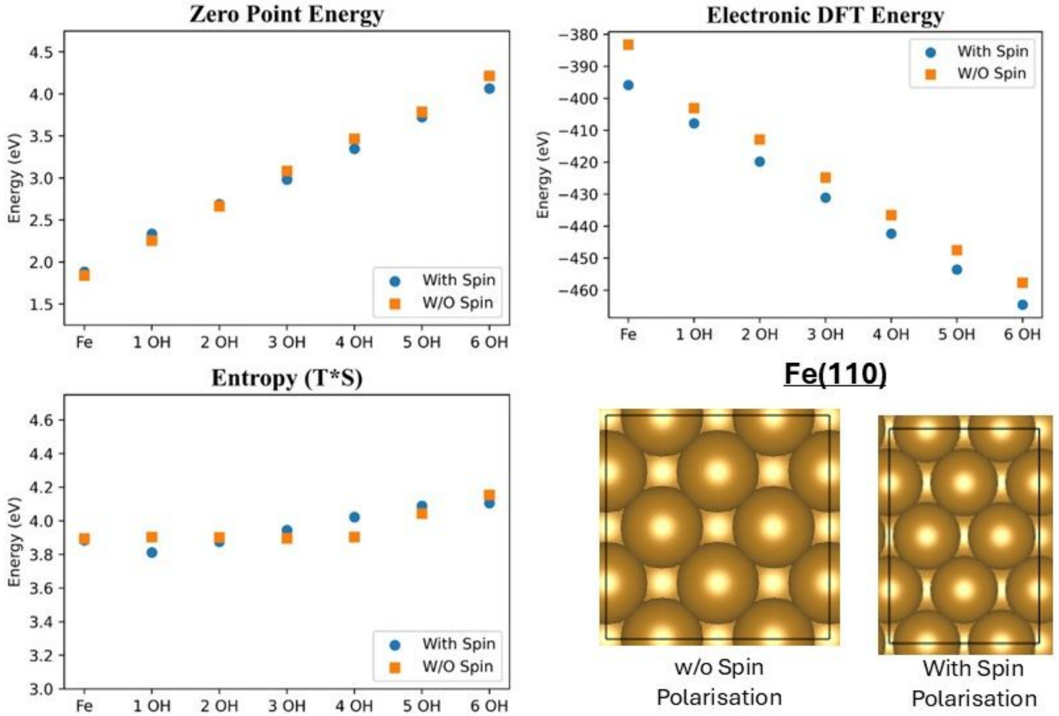

*Figure S3: The DFT energy, ZPE, and entropy (T*S) energy values of $^{*}OH$ adsorption at different coverage using spin-polarised and non-spin-polarised calculations with the geometry of the Fe(110) surface.*

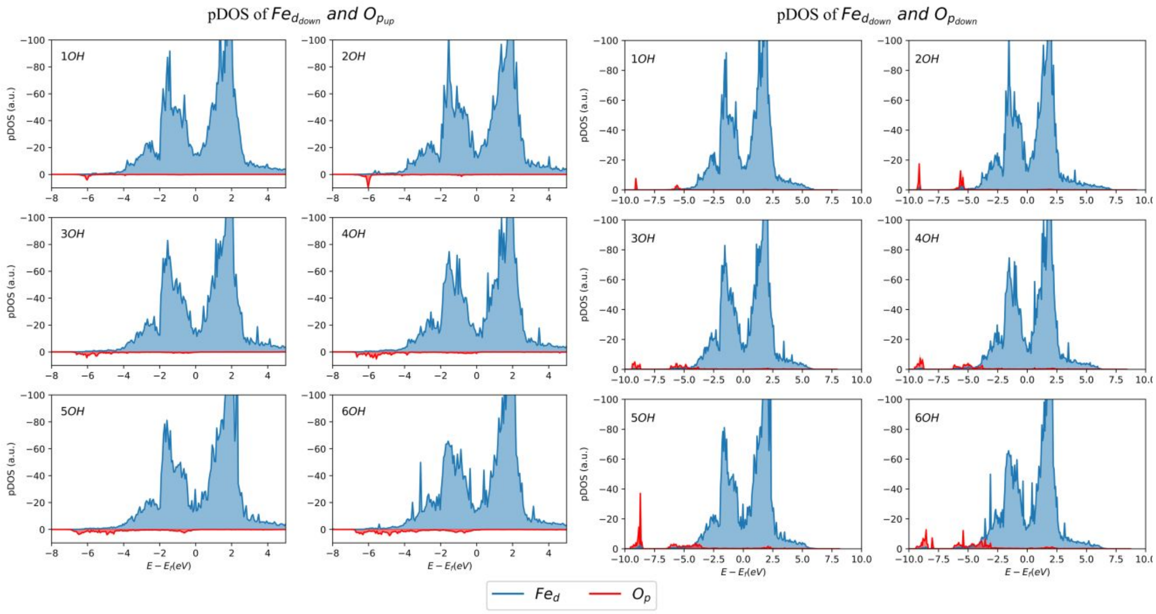

Figure S4: The pDOS of Fe-d_{down} spin states with O_p at different surface coverage of $^*OH$ on the Fe(110) surface.

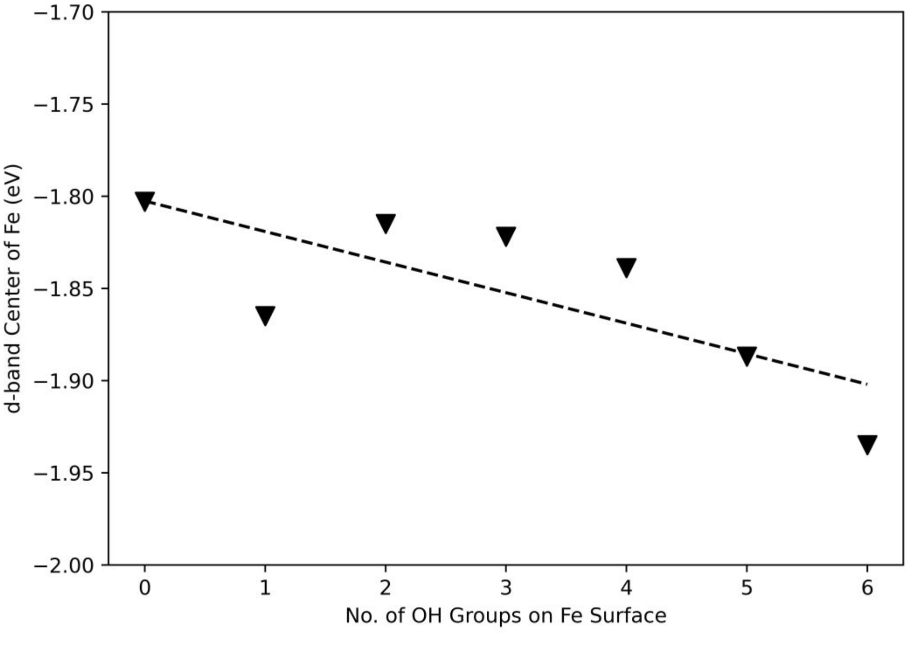

Figure S5: The d-band center (ε_d) values of Fe at different surface coverage of OH on the surface.

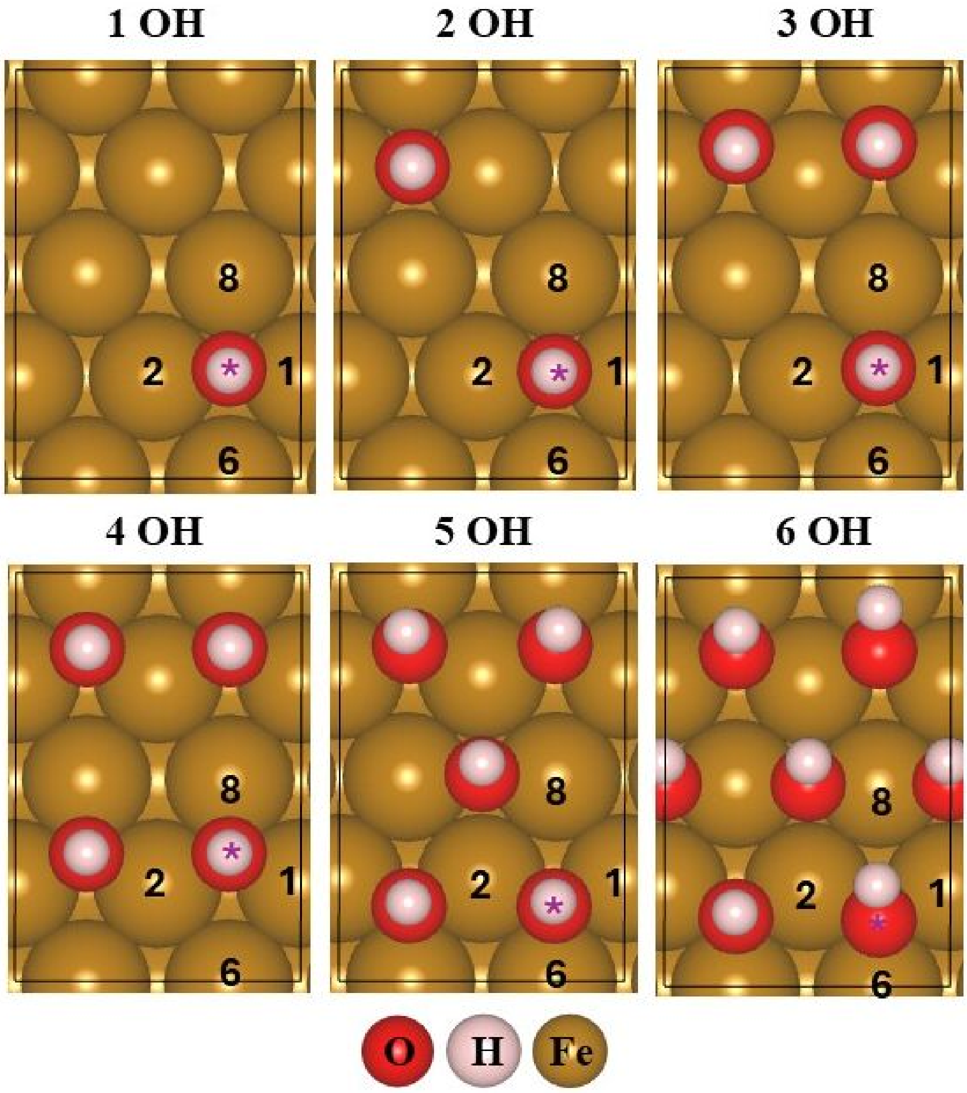

*Figure S6: The optimised geometries of *OH on the Fe(110) surface at different coverages with labelling of Fe-O atoms considered for the ICOHP analysis.*

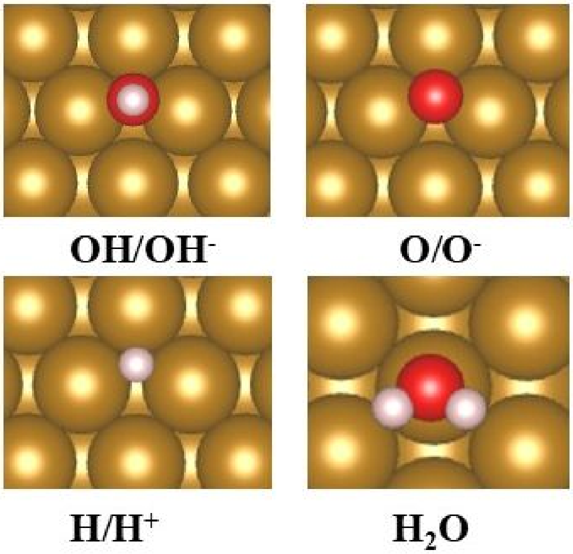

Figure S7: The optimised geometries of adsorbates are given in Table 2 on the Fe(110) surface.

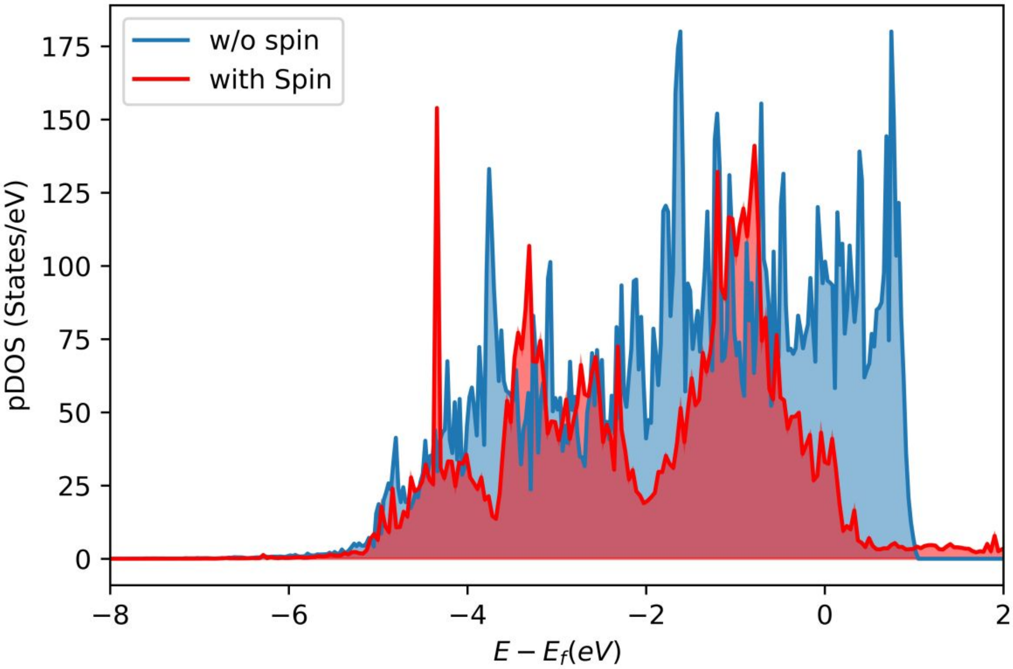

Figure S8: The partial density of states (pDOS) of the d orbital of the Fe(110) surface with and without spin-polarisation.

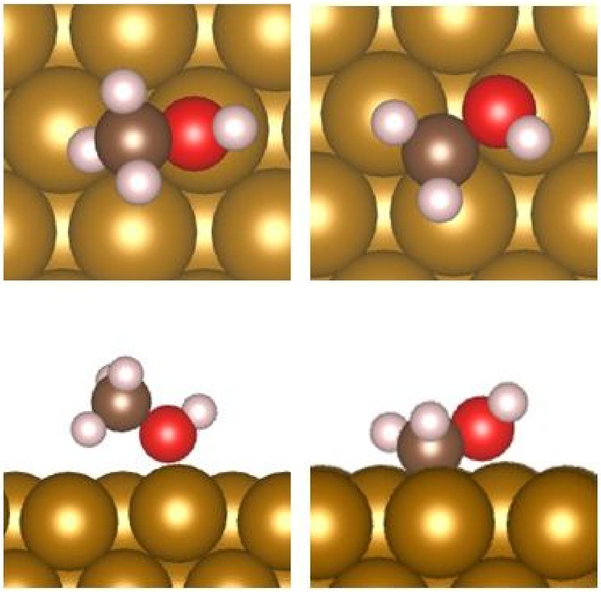

CH_3OH CH_2OH

Figure S9: The optimised geometries of CH_3OH and CH_2OH on the Fe(110) surface.

3. Calculating the reduction potential of *OH

1. $^*OH + \bar{e} \rightarrow \; ^*OH^-$

2. $H^+_{aq} + \bar{e} \rightarrow \frac{1}{2}H_{2aq}$

3. $^*OH + H^+_{aq} \rightarrow \; ^*OH^- + \frac{1}{2}H_{2aq}$

$$\Delta G(3)^0 = G^0(\,^*OH^-) + \frac{1}{2}G^0\left(H_{2aq}\right) - G^0(\,^*OH) - G^0(H^+_{aq})$$

4. $OH^{\cdot}_{aq} + \bar{e} \rightarrow OH^-_{aq}$

5. $OH^{\cdot}_{aq} + H^+_{aq} \rightarrow OH^-_{aq} + \frac{1}{2}H_{2aq}$

$$\Delta G(5)^0 = G^0\left(OH^-_{aq}\right) + \frac{1}{2}G^0\left(H_{2aq}\right) - G^0\left(OH_{aq}\right) - G^0(H^+_{aq})$$

6. $\Delta G(5)^0 - \Delta G(3)^0 = G^0\left(OH^-_{aq}\right) - G^0(\,^*OH^-) - G^0(\,*\,) - G^0\left(OH_{aq}\right) + G^0(\,^*OH) + G^0$ $(\,*\,)$

$$= G^0_{ads}(OH^{\cdot}) - G^0_{ads}(OH^{\cdot})$$

Reaction 3 is reaction 1 minus reaction 2, and reaction 5 is reaction 4 minus reaction 2. In equation 6, $G^0(*)$ was added and subtracted to obtain the adsorption energies.

$G^0(N)$ – free energy of reaction N

$G^0(X)$ – free energy of X

$G^0(*)$ – free energy of the plain surface

$G^0_{ads}(X)$ – free energy of adsorption of X.

The relationship between the two physical quantities, ΔG° and E°, is given by the equation ΔG° = –nFE°, where ΔG° is the standard Gibbs free energy change, n is the number of electrons transferred, F is the Faraday constant, and E° is the standard electrode potential. Since ΔG° values are in eV, and n = 1, multiplying Eq. 6 by -1 gives the expression for surface reduction potential:

$E^0(3) – E^0(5) = G^0_{ads}(^{\cdot}OH) - G^0_{ads}(OH^-)$

$E^0(3) = E^0(5) + G^0_{ads}(^{\cdot}OH) - G^0_{ads}(OH^-)$

$E^0(3)$ =1.90 – 3.38 +1.63 = 0.15 V

The redox potential of reaction 3 ($E^0(3)$) is the redox potential of reaction 1 vs. SHE. This is the reduction potential of adsorbed OH. In the same way, the redox potential of reaction 5 ($E^0(5)$) is the redox potential of reaction 4 vs. SHE; this is the reduction potential $^{\cdot}$OH radicals in aqueous solution.

4. Structural Information

The optimised geometries of computed structures are accessible at https://doi.org/10.5281/zenodo.18134500

The files can be visualised using packages such as VESTA.
